# Monitoring of patients with microdialysis following pancreaticoduodenectomy—the MINIMUM study: study protocol for a randomized controlled trial

**DOI:** 10.1186/s13063-021-05221-9

**Published:** 2021-05-07

**Authors:** Espen Lindholm, Nil Ekiz, Tor Inge Tønnessen

**Affiliations:** 1 Department of Anesthesiology, Division of Emergencies and Critical Care, Oslo University Hospital, Rikshospitalet, Postboks 4950 Nydalen, 0424 Oslo, Norway; 2 Department of Anesthesiology, Clinic of Surgery, Vestfold Hospital Trust, 3103 Tønsberg, Norway; 3 Institute of Clinical Medicine, University of Oslo, 0316 Oslo, Norway

**Keywords:** Postoperative pancreatic fistula, Anastomotic leakage, Pancreatoduodenectomy, Microdialysis

## Abstract

**Background:**

Postoperative pancreatic fistula after pancreatoduodenectomy is a much-feared complication associated with substantial mortality and morbidity. The current standard for diagnosing postoperative pancreatic fistula, besides routine clinical examination, include radiological examinations, analysis of pancreatic drain amylase activity, and routine blood samples. Another promising method is by intraperitoneal microdialysis to monitor intraperitoneal metabolites measured at the pancreaticojejunostomy, thereby detecting what occurs locally, before chemical events can be reflected as measurable changes in systemic blood levels.

**Methods:**

The MINIMUM study is a prospective, randomized, controlled, single center enrolling 200 patients scheduled for open pancreatoduodenectomy comparing the microdialysis method to the “standard of care.” Half of the included patients will be randomized to receive an intraperitoneal microdialysis catheter implanted at the end of surgery and will be monitored by microdialysis as an additional monitoring tool. The other half of the patients will not receive a microdialysis catheter and will be monitored according to the current standard of care. The primary objective is to evaluate if the microdialysis method can reduce the total length of stay at the hospital. Secondary endpoints are the frequency of complications, length of stay at the hospital at our institution, catheter malfunction, number of infections and bleeding episodes caused by the microdialysis catheter, patient-reported quality of life and pain, and cost per patient undergoing pancreatoduodenectomy. The patients will be randomized in a 1:1 ratio.

**Discussion:**

Intraabdominal microdialysis could potentially reduce morbidity and mortality after pancreatoduodenectomy. Furthermore, there is a great potential for shortening the in-hospital length of stay and reducing the financial aspect considerably. This study may potentially open the possibility for using microdialysis as standard monitoring in patients undergoing pancreatoduodenectomy. The hypothesis is that the microdialysis method compared to “standard care” will reduce the total length of hospital stay.

**Trial registration:**

Clinicaltrials.gov (NCT03631173). Registered on 7 September 2018 under the name: “Monitoring of patients With Microdialysis Following Pancreaticoduodenectomy”. Based on protocol version 19-1, dated 15th January 2019.

**Supplementary Information:**

The online version contains supplementary material available at 10.1186/s13063-021-05221-9.

## Background and rationale

### Pancreatoduodenectomy and postoperative pancreatic fistula (POPF)

Pancreatoduodenectomy is the treatment of choice for tumors of the periampullary region. Due to its high-risk nature, pancreatoduodenectomy has become a routine procedure in specialized high-volume centers, and mortality has decreased significantly over the last decades. To achieve better results, several factors have been identified and improved upon; pre-and postoperative management, appropriate selection of patients, improved surgical tools and skills, and the development of multidisciplinary teams [1, 2]. However, even if mortality is less than 3–5% in experienced hands, the overall morbidity rate is still high—from 30% to 50%—leading to prolonged in-hospital stay and increased costs [3]. There are 3 different anastomoses (surgical connection between two structures) with the potential for a leak post-pancreatoduodenectomy; the gastrojejunostomy, choledochojejunostomy, and the pancreaticojejunostomy. An anastomotic leakage after pancreatoduodenectomy is a feared complication with substantial mortality and morbidity. Formation of a postoperative pancreatic fistula (POPF) is a serious complication. Pancreatic secretions with its eroding enzymes like proteases and lipases erode surrounding tissues and lead to partial or complete anastomotic dehiscence and break-down of surrounding tissue. In addition, the leakage of pancreatic secretions, through a fistula in the pancreatico-enteric anastomosis can cause inflammation and auto-destruction of the peripancreatic and retroperitoneal tissues as well as the surrounding vessels and viscera. Subsequently, this can lead to delayed gastric emptying, biliary leakage, development of vascular erosions and hemorrhage, and lastly intra-abdominal abscesses. These may all lead to sepsis, shock, multiorgan failure, and death [3]. The incidence of POPF is reported to lie between 0% and 36% in older reports, but it tends to be much lower in high-volume centers and newer reports. Still, the most recent incidence of POPF after pancreatoduodenectomy is in the range of 20–25% [4–7]. The largest contributor to the postoperative mortality is due to these complications.

The treatment of POPF can be difficult and management may range from simple observation with or without percutaneous drainage, to the urgent reoperation and management of abdominal sepsis with organ failure and prolonged intensive care [7]. Clinically, POPF is divided into two severity grades (B or C) [8]. The risk of death and severe morbidity increases considerably from a biochemical leakage to a POPF grade C. Studies have shown that the cost associated with a patient with a fistula is 1.3–6 times that of a patient with no complications after pancreatoduodenectomy [9, 10].

The current standard for diagnosing POPF involves, alongside bedside clinical examination, the analysis of pancreatic amylase activity from intraabdominal drains and routine blood samples, and radiological imaging if pathology is suspected. A novel method is intraperitoneal microdialysis to monitor intraperitoneal metabolites (glycerol, lactate, pyruvate, and glucose) measured at the pancreaticojejunostomy.

### Microdialysis

A microdialysis catheter is constructed as a concentric tube where a perfusion fluid enters through an inner tube, flows to its distal end, exits the tube, where the exchange between the intraperitoneal fluid and the perfusion fluid takes place. The perfusate equilibrates with molecules in the intraperitoneal fluid. Thereafter the fluid enters the space between the inner tube and the outer dialysis membrane and the direction of flow is now reversed. The fluid moves toward the proximal end of the catheter and is finally collected in a microvial. Microdialysis is a technique to monitor the chemistry of the extracellular space in near real-time, thereby detecting what occurs locally, before chemical events can be reflected as measurable changes in systemic blood levels.

The microdialysis technique monitors substances produced by the products of cell metabolism. Microdialysis is a technique that has been utilized extensively in fields of plastic surgery and neurosurgery among others. Few studies with intraperitoneal microdialysis have been performed, but these are few in numbers and include few patients [11–17]. A small number of studies have investigated the use of microdialysis related to leakage from anastomosis between bile and pancreatic ducts. Intraperitoneal microdialysis will be investigated as a method to detect anastomotic leakage earlier than clinical signs appear. End-organ monitoring with microdialysis catheters with regular measurements of lactate, pyruvate, glucose, and glycerol has been shown to be a sensitive and safe method of detection of circulatory disorder in, i.e., liver transplants and colon anastomoses [16, 18]. During periods of ischemia, metabolic changes can be observed in the perianastomotic areas by increases in lactate, reduction of glucose, and an increase of lactate-to-pyruvate (L/P) ratio. A study by Ansorge et al. [19] showed that patients with a POPF had higher intraperitoneal glycerol concentrations and lactate/pyruvate ratios, and lower glucose concentrations. Other studies [11–28] have demonstrated that intraperitoneal microdialysis is a promising tool for the early detection of anastomotic leakage and intraabdominal pathology in patients undergoing abdominal surgery, but these studies have been small and not randomized. As to the best of our knowledge, there are currently no randomized studies that have looked at microdialysis as a method of detecting anastomotic leakage after pancreatic surgery. Monitoring immediately after surgery in patients undergoing pancreatoduodenectomy may provide an advantage by allowing for prompt interventions. Consequently, this may reduce the total length of stay (LOS) at the hospital. Therefore, further adequately powered randomized trials are needed.

## Methods/design

Our hypothesis is that the microdialysis method compared to “standard care” will reduce the total length of hospital stay.

### Objectives and endpoints

Our primary objective is to evaluate if the microdialysis method will reduce the total LOS at the hospital in patients undergoing pancreatoduodenectomy. The primary and secondary objectives combined with the primary, secondary, and other explorative endpoints and how they are assessed are presented in Table 1.

**Table 1:** Primary and secondary objective. Primary, secondary, and other explorative endpoints and how they are assessed

Objectives		Endpoints	Assessments
Primary	To evaluate if the microdialysis method will reduce the total length of stay at the hospital(s).	Number of days/hours from end of surgery to hospital discharge from primary hospital plus number of days/hours for subsequent admissions with a diagnosis associated with the primary surgery at any hospital	Hours/days from the initial operation (end of surgery) to hospital discharge. All hospitals admitting the patient are included, also transferred hospitals. From electronic patient records.
Secondary 1	To evaluate predictive score systems for POPF	Occurrence of POPF	POPF defined according to the definition of the ISGPF. Graded into “biochemical”, B or C. From medical record, CT-scans. The following risk factors will be assessed: Age, gender, smoking history—current and package years, preoperative BMI, weight loss, Intraabdominal fat thickness, pancreatitis history, relation to portal vein to tumor, primary diagnosis, radiological (assessed by CT-scan) PD width, intraoperative PD width, intraoperative blood loss, pancreatic texture, pancreatic fat, pancreatic fibrosis, drain amylase. From preoperative examination, medical record, CT-scan, during surgery and postoperative examinations.
Secondary 2	To evaluate if microdialysis data contribute to reduced length of stay at the primary hospital and ICU	Length of stay at the primary hospital Length of stay at the ICU	Number of days/hours from end of initial operation to primary hospital discharge and hours admitted at the ICU. From electronic patient records.
Secondary 3	To evaluate if there is a special pattern of inflammatory markers in the microdialysate and serum in patients with/without POPF	Concentration of inflammatory markers	From laboratory analysis
Secondary 4	To evaluate the reliability and complications using microdialysis catheter CMA 65	Occurrence of catheter malfunction Occurrence of bleedings and infections	Daily check of the microdialysis catheter is functioning. Assessments of bleeding which affects circulatory parameter (development of circulatory shock or need of transfusion) and infection
Secondary 5	To evaluate patient quality of life and pain	Overall score and sub-scale scores of patient-reported questionnaires	Two patient-reported questionnaires: “Abdominal surgery impact scale” will be gathered with patient-reported McGill Pain Questionnaire-2 (SF-MPQ-2) preoperatively, at POD3 ± 1 day and at discharge ± 2 days from primary hospital + 30 and 90 days after surgery.
Secondary 6	To compare hospital costs of using microdialysisis versus “standard of care”	Number of Euros per patient undergoing PD based on microdialysis costs, length of stay (ICU and inpatient stay), reoperations, and postoperative complications	From medical records, procedures noted in electronic patient records, and radiological electronic patient records.
Exploratory	To compare other endpoints between patient with and without a microdialysis catheter	Hours from end of surgery to diagnosis of postoperative pancreatic AL (grades B and C).	Time as noted in electronic patient records.
		Total quantity (μg/mg) of vasoactive medications during surgery	Amount during surgery where the PD was performed. Derived from electronic patient records.
		Fluid balance - total iv volume administered and total diuresis	Diuresis and amount of fluid given iv during surgery and postoperatively until discharge from the hospital where the PD was performed. Derived from electronic patient records.
		Number of patients with biliary fistula	Biliary fistula defined according to the definition of the International Study Group of Liver Surgery (ISGLS). Graded into A, B, or C. From medical record, CT-scans.
		Number of patients with gastro-enteric AL	Gastro-enteric AL. From medical record, CT-scans.
		Pancreatic amylase and bilirubin concentrations in drainage fluid and in serum.	Analysis of drainage fluid and serum
		White blood cell count, C-reactive protein; concentrations	Laboratory data
		Number of patients with postoperative complications during total hospital stay in total and per complication	Defined by the modified Clavien-Dindo classification, from medical record, radiological examinations, and electronic patient records.
		Patient’s discharge disposition	From electronic patient records.

*AL* anastomotic leakage, *BMI* body mass index, *CT* computed tomography, *ICU* intensive care unit, *ISGLS* International Study Group of Liver Surgery, *ISGPF* International Study Group on Pancreatic Fistula, *iv* intravenous, *PD* pancreatoduodenectomy, *POD* postoperative day, *POPF* postoperative pancreatic fistula

### Inclusion and exclusion criteria

The patients must meet all of the following inclusion criteria at the time of enrollment

- Must be scheduled for a pancreatoduodenectomy
- Must be ≥ 18 years
- Must be able to give written, signed, and informed consent
- The Investigator must ascertain that the patient is able to understand, comply and follow the instructions needed to successfully participate in this trial

The following must not be present at the time of enrollment

- Allergy to the perfusion fluid Voluven® (Fresenius Kabi AS, Halden, Norway)
- Allergy to the contrast agent given during CT scan
- Any medical- or other condition that the surgeon deems sufficient to make the patient unfit for participation in the trial
- Participation in other interventional clinical studies interfering with the current study
- Pregnant
- Any infectious disease that makes microdialysis analysis impossible to carry out
  No inclusion/exclusion criteria for the surgeon exist.

### Design, enrollment, inclusion, and randomization

The MINIMUM study is a 2-armed, single-center, randomized, parallel-group trial with an equivalence framework. The study population is patients scheduled for open pancreatoduodenectomy at Oslo University Hospital – Rikshospitalet, Norway. This center normally performs 130 open pancreatoduodenectomy each year and MINIMUM seeks to enroll 200 consecutive patients scheduled for open pancreatoduodenectomy. Half of the included patients (intervention group) will be randomized to receive an intraperitoneal microdialysis catheter inserted at the end of surgery and will postoperatively be monitored by microdialysis. The surgeon will have full access to the microdialysis results at any time during the study period. The surgeon may intervene based solely on the patient’s symptoms and signs, plus predetermined values of the microdialysis measurements. The other half of the patients (control group) will not receive a microdialysis catheter. The patients are monitored according to the current standard of care and the surgeon may intervene based only on symptoms and signs. The only difference between the two groups is that the treating surgeon in the intervention group will implant a microdialysis catheter close to the pancreaticoenterostomy. Once implanted, the microdialysis results will be available to the treating team, and these results can be included into the assessment and management of the patient. Group assignments will be determined by an online randomization system; (Viedoc™) developed by PCG Solutions (S:t Persgatan 6, Uppsala, Sweden). Screening and enrollment of patients will be performed by the investigators. All the patients will be included minimum 1 day prior to surgery. The study will be conducted according to the Helsinki declaration. A special combined patient information leaflet and consent form has been developed for the MINIMUM study. Patients will be recruited from the operation planning program on site. After informed consent has been obtained by the principal investigator or co-investigator, the patients will be included. The trial will be conducted in accordance with the Consolidated Standards of Reporting Trials (CONSORT) Statement.

The study has been approved by the institutional review board of Division of Emergencies and Critical Care, Oslo University Hospital, the regional ethics committee in Norway (Regional Committees for Medical and Health Research Ethics – Additional files 3 and 4) and has been registered at clinicaltrials.gov (NCT03631173). Insurance policies for all patients are obtained by the Norwegian National System of Patient Injury Compensation.

### Blinding

In this study, there will be no blinding of the patients or the health care providers in regards to the microdialysis catheter or the results from the bedside microdialysis results. The group affiliation is obvious for the different caregivers because of the highly visible microdialysis catheter and frequent analyses of the microdialysate.

### Participant timeline

Randomization started in April 2019 at Oslo University Hospital and will last for 2 years. The Assessment Schedule is presented in Table 2.

**Table 2:** Assessment Schedule

	Study period						
Event	Pre-admission	Pre-operative	Intra-operative	Post-operative			
				During admission	At discharge	30th POD	90th POD
**Eligibility screen**	≥ 1 day prior surgery						
**Information - signed informed consent**	≥ 1 day prior surgery						
**Inclusion**	X						
**Randomization**			X				
**Intervention - microdialysis**			X	X			
**Control (standard care)**			X	X			
**Start eCRF**	X						
**Premedication**		X					
**Epidural**		X					
**Arterial catheter**		X					
**Central venous line**		X					
**Targeting anesthesia**			SpO_2_ ≥ 93%. BPmap ≥ 60 mmHg. Body temperature ≥ 36 °C. Ventilatation with 6–8 ml/kg PBW				
**Pantoperazole 40 mg iv**				Administered daily for 7 days			
**Assessments**							
**Patient characteristics**	Gender, age, height, weight, BMI, blood pressure, heart rate, SpO2						
**SOFA-score**	X			Daily until POD 10 if applicable			
**Weight loss last 6 months**	X						
**Concomitant medication**	X						
**Smoking history**	Current, pack years						
**Medical history**	Including pancreatitis						
**Preoperative chemotherapy**	X						
**Preoperative CT scan**		X					
** Intraabdominal fat thickness**		X					
** The relation to portal vein to tumor**		X					
** Width of pancreatic duct**		X					
** Width of pancreas**		X					
** Current diseases**	Histology – differentiation of tumors and TNM staging	X					
**Quality of life questionnaire - Abdominal surgery Impact scale**	> 1 day before surgery. After inclusion but before randomization			POD 3	X	X	X
**Short-form McGill Pain Questionnaire-2 (SF-MPQ-2)**	> 1 days before surgery. After inclusion but before randomization			POD 3	X	X	X
**Blood samples**	Hb, Trc, WBC, ASAT, ALAT, GGT, ALP, LD, Amylase, Bilirubin, Creatinine, Urea, GFR, CRP, a panel of inflammatory markers, s-lactate, and arterial blood gas.			Daily at 08.00 am: Hb, Trc, WBC, Amylase, ASAT, ALAT, GGT, LD, ALP, Bilirubin, Creatinine, GFR, UREA, CRP, a panel of inflammatory markers, s-lactate, arterial blood gas.			
**iv fluid**			X				
**Urine output**			X				
**Blood loss**			X				
**Transfusion**			X	X			
**Use of vasoactive medicaments**			X				
**Intraoperative PD width**			X				
**Intraoperative pancreatic consistency**			X				
**Histological assessment**							
**Pancreatic fat**			X				
**Pancreatic fibrosis**			X				
**Drain tube**			Insertion	Cessation POD 1–3 (or more) unless the effluent is bile, enteric stained or turbid or depending on microdialysis concentrations			
**Amylase and bilirubin in drain fluid**				At POD 1–3 and on indication thereafter if drain still in place			
**Pantoperazole 40 mg iv**				Administered daily for 7 days			
**Sampling/analyzing microdialysate**				Every hour in the first 24 h. Thereafter, every two hours until POD 2. From POD 2: every 4th hour until discharge; Pyruvate, lactate, glucose, glycerol			
**Collecting microdialysate for cytokines**				Twice daily (≈ 08.00 am and 08.00 pm)			
**Microdialysis catheter function and duration**			X	X			
**Abdominal CT scan during admission(s)**				POD 2 in patient with high microdialysate concentrations in three consecutive microdialysate samples and/or at the surgeon's discretion	at the surgeons discretion	at the surgeons discretion	at the surgeons discretion
**Postoperative complications - Clavien-Dindo classification ≥ 2**				X	X	X	X
**Time from end of surgery to a diagnosed anastomosis leakage, if applicable**				X	X	X	X
**Type of complications**				X	X	X	X
**Type of procedures due to complications**				X	X	X	X
**Cost of complications**				X	X	X	X
**Transfer(s) to ICU**				X	X	X	X
**LOS at primary hospital and ICU**				X	X	X	X
**LOS at secondary hospital**						X	X
**Mortality**			X	X	X	X	X

*ALAT* alanine aminotransferase, *ALP* alkaline phosphatase, *ASAT* aspartate aminotransferase, *BMI* body mass index, *CRP* c*-*reactive protein, *CT* computed tomography, *GFR* glomerular filtration rate, *GGT* gamma*-*glutamyl transferase, *Hb* hemoglobin, *ICU* intensive care unit, *LD* lactate dehydrogenase, *LOS* length of stay, *PBW* predicted body weight, *PD* pancreatic duct, *POD* postoperative day, *SpO2* peripheral oxygen saturation, *TNM* tumor-nodule-metastasis, *Trc* platelets, *WBC* white blood cells

### Miscellaneous data to be collected

All data will be plotted in a password-protected web-based eCRF (Viedoc™, PCG Solutions, S:t Persgatan 6, Uppsala, Sweden) consecutively by the study team (investigators and study nurses).

Baseline demographic data, current medication, and the presence of any chronic diseases will be collected. Intraoperative data and postoperative complications, as defined by the modified Clavien-Dindo classification, will be recorded until POD 90. Total hospital stay + LOS at the hospital/ICU, pre- and postoperative CT-scan results, mode of nutrition, and any transfusion requirements will also be recorded. Sequential Organ Failure Assessment scoring (SOFA) will be performed preoperatively, and up to POD 10 if the patient is still admitted at the primary hospital. Also, the investigators will calculate the financial burden related to ICU stay, inpatient stay at the hospital, readmissions, reoperations, and (expensive) treatment procedures and examinations performed as a consequence of a POPF-development.

### Microdialysis catheter and microdialysis analysis

Our research group has previously shown that microdialysis catheters with pore size for particles up to 100 kDa collect cytokines and complement factors, consequently, CMA 65 is used in this study [28]. The catheter is flexible and allows for the continuous monitoring and detection of local metabolic changes in the gastrointestinal tract. The catheter has a shaft length of 180 mm with a 30 mm membrane made of polyarylethersulfone. The diameter is 0.9 mm. The microdialysis catheter is tested for functionality before implantation. Before skin closure, the catheters will be implanted through a percutaneous hypodermic needle and affixed with an absorbable suture to surrounding connective tissue in close vicinity (< 1 cm) to the pancreaticojejunostomy. An epidermal suture will be used to minimize the risk of unintentional dislodgement. The catheters will be kept until the patient is discharged from the primary hospital or the catheter malfunctions.

The catheter is connected to a small battery-driven syringe pump (CMA 107, M Dialysis AB) and perfused by hydroxyethyl starch solution 130/0.4 (Voluven®, Fresenius Kabi AS, Halden, Norway) pumped at a rate of 1 μL/min as dialysis solution. The microdialysate will be collected in microvials which will be changed and analyzed hourly for metabolic parameters and cytokines. The samples thereafter will be analyzed for lactate, pyruvate, glucose, and glycerol with ISCUSflex Microdialysis Analyzer® (M Dialysis AB, Johanneshov, Sweden) by trained nurses/physicians in the following manner:

- Every hour for the first 24 h.
- Thereafter, every 2 h as long as the microdialysis catheter still functions. With the exception of the initial 24 h, nighttime samples will be done at 0.00, 4.00, and 8.00 am.
- After POD 2 at 0.00 am: If the lactate/pyruvate-ratio increases > 50 combined with lactate > 4 mmol/L AND/OR glycerol increases > 400 μmol/L, the next two samples will be performed with an interval of 2 h (in place of every 4 h at night) to confirm or refute a pathological process.

Twice daily (≈ 08.00 am and 08.00 pm), samples from the microvials will be kept, and frozen to − 80 °C for later examination of cytokines and complement factors.

### Blood samples

Daily blood samples will be obtained daily until discharge as described below. Additional blood will be obtained daily which will be processed within 1 h after collection by centrifugation at 2500 RPM or 10 min at 4 °C, and the supernatant thereof will be separated. This will thereafter be stored at − 80 °C for later examination of cytokines and complement factors at the Immunological institute, Oslo University Hospital, Norway. A fraction of the included patients will be selected for analysis of inflammatory markers in the frozen serum and microdialysate. .

### Abdominal drain fluid

Measurements of amylase activity and bilirubin from abdominal drains will be performed postoperatively in all patients.

### Patient-reported outcome measures

We will collect two self-report questionnaires. The patient questionnaire “Abdominal surgery impact scale” will be collected together with a self-reported McGill Pain Questionnaire-2 (SF-MPQ-2) preoperatively, at POD 3, at discharge, and then at POD 30 and 90. The Abdominal Surgery Impact Scale was translated into Norwegian according to the forward and backward translation method used by Tsang et al. [29].

### Assessment and management of patient based on microdialysis results

**Fig. 1:**
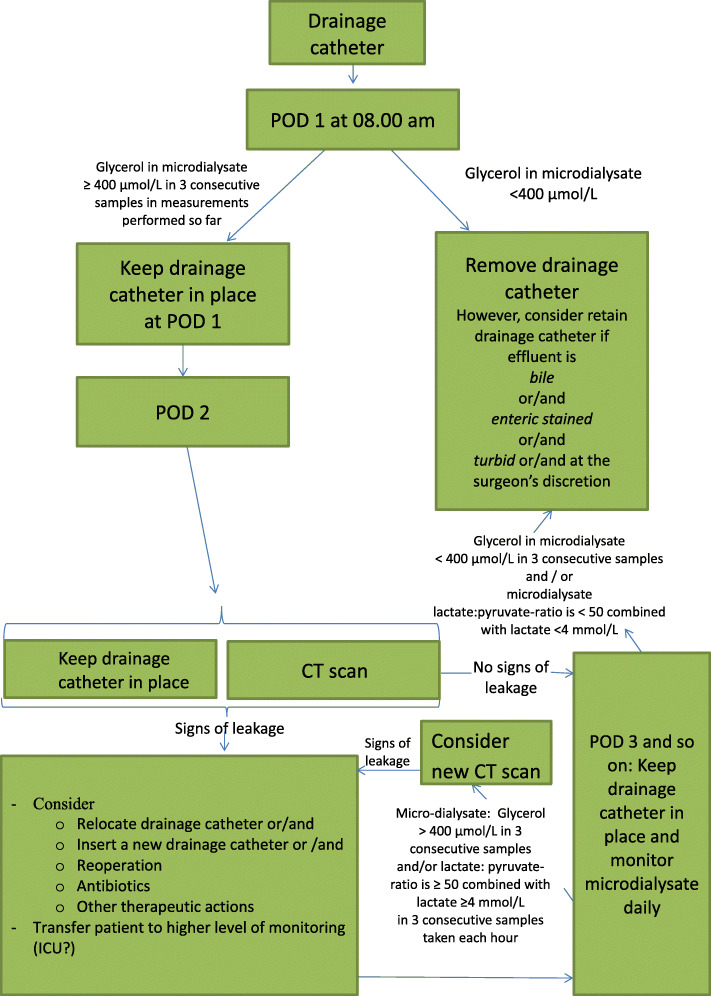
Algorithm for assessment of patients with microdialysis. Postoperative treatment algorithm - Assessment and management of patient based on microdialysis results. CT, computer tomography; ICU, intensive care unit; L, liter; POD, postoperative day

All patients in the intervention group with microdialysis glycerol > 400 μmol/ L during the first 48 h in three consecutive samples in the microdialysate will have an abdominal CT scan on POD 2. This examination will be reviewed by two senior radiologists allocated to this study and assessed according to the preexisting definitions of an abscess or radiologically visible anastomotic disruption. In the case of positive radiological findings, a subsequent CT scan will be performed according to algorithm (Fig. 1) and is described below. In the case where there is a clinical suspicion of an anastomotic leakage regardless of microdialysate values, an abdominal CT scan may be performed at the discretion of the surgeon at any time during the hospital stay, independent of microdialysate values. The CT scan will initially be assessed by the surgeon and the radiologist on call, but will also later be assessed independently by the two radiologists assigned to this study.

Assessment of glycerol, lactate, and pyruvate concentration is done by the surgeon.

- If the glycerol level is ≥ 400 μmol/l in 3 consecutive samples at POD1, the abdominal drain is kept to POD 2, and a CT scan is performed. If a leakage is confirmed, treatment is initiated in the following manner:

Consider

- Optimizing the location of the abdominal drain and/or
- Insertion of a second abdominal drain and/or
- Relaparotomy
- IV antibiotics
- Other therapeutic actions
- Transferring the patient to a higher level of care

The patient will be further assessed with clinical signs, measurement of abdominal drain output, and microdialysis values. A repeat CT (or ultrasound/ MRI) scan later in the course will be performed if the microdialysate lactate-to-pyruvate ratio is ≥ 50 combined with a lactate ≥ 4 mmol/L in 3 consecutive samples (irrespective of the time since end of surgery. If a leakage is diagnosed, the treatment described above is initiated. A repeat CT scan in a patient diagnosed with a leakage is performed on the basis of new microdialysis values (both absolute levels, and the trends thereof), the clinical condition of the patient, amylase activities, and bilirubin measured in abdominal drains.

The decision to perform a new scan and how often this will be appropriate is an interdisciplinary assessment between the surgeons and the anesthesiologist. Abdominal drains are removed later in the course if microdialysate lactate-to-pyruvate ratio is < 50, combined with a lactate < 4 mmol/L in three consecutive samples and there is no other reason to postpone their cessation (no signs of bile, chylous or enteric stained fluid and/or at the surgeon’s discretion).

b) If glycerol is persistently < 400 μmol/l within the first 24 h the abdominal drains are removed at POD 1 (prerequisite: if no signs of bile, chylous or enteric stained fluid or at the surgeon’s discretion). After this, the patients are assessed and microdialysate results are reviewed. If microdialysate glycerol is persistently < 400 μmol/l over several PODs and the lactate-to-pyruvate ratio is < 50 combined with a lactate < 4 mmol/L, the patient is further mobilized and subsequently discharged. If the glycerol increases ≥ 400 μmol/l in 3 consecutive samples at a later stage, the patient is examined, and CT scan (or ultrasound/MRI) is performed (at the discretion of the surgeon). Further intervention undertaken will be as described above.

To improve adherence to the intervention protocols, the researchers arranged an introductory- and regular follow-up meetings with the surgical team during the ongoing trial. An allocated researcher was assigned for the daily contact with the surgical team. Study newsletters and bedside training for the ward nurses were regular events during the ongoing trial. To ensure participant adherence, the patients received daily follow-up by the research team during the hospital stay, and subsequently by telephone interview at 30 and 90 days after surgery.

### Data monitoring

Monitoring the study will ensure the quality of the clinical investigation and will be performed in the MINIMUM study. The study will be monitored by “The Clinical Trials Unit,” an independent entity to the sponsor. “Monitoring” means that eCRFs will be audited to make sure that they have been completed, correctly registered and that the data are within expected values. If there are any elements of uncertainty, discrepancies in the data, missing data, or any errors are detected, the monitor will add queries into the eCRF, addressed to the investigator responsible for the patients. The monitor will track and follow these queries in cooperation with the local investigator and PI until a resolution of these queries is achieved. The monitor will use 4 days of monitoring during the study period.

### Statistical considerations

The following assumptions underlie the sample size calculations

- The distribution of the primary endpoint is according to Pratt et al. [9]
- The effect of the intervention is measured as a reduction in median time in total hospital stay

With data collected from 35 patients undergoing the same procedure at Oslo university hospital and the median LOS in these patients, and with an assumed left censoring at 4 days (assuming that it is not possible with an admission length of less than 4 days), Weibull estimates and Log-logistic estimates were performed. A plot with the different models together with the empirical cumulative probability plot was designed and the Log-logistic model gave the best fit. However, the fit is best for the observations with short LOS, while the fit in the tail was not so good. A slight shift of 0.5 in the shape parameter gave a better fit in the tail. With the heavier tail log-logistic distribution and a reduction in median time to discharge of a clinical realistic 1.5 days, a simulation analysis with 4000 draws gave the following result:

**Table:** 

Efficacy	Power	Sample size per group
-1 day(s)	0.818	155
-1 day(s)	0.909	202
-1.5 day(s)	0.814	58
-1.5 day(s)	0.932	84

In the MINIMUM study, we assume a reduction in total hospital stay ~ 1.5 days. With a significance of 0.05 and a power of 0.93, we will randomize 100 patients in each group considering that ~ 15% of the patients will not have a pancreaticoduodenectomy performed. If this number appears to be higher than the first estimate, the sample size needs to be recalculated.

The statistical analysis plan will be developed and finalized before database lock and will describe the participant populations to be included in the analyses, along with procedures for the accounting of missing, unused, and spurious data. The statistical analysis plan will be developed partly using blinded data to check for assumptions. Blinding is obtained by exchanging the treatment variable with a random allocation variable prior to any results. No unblinded analyses will be performed prior to the finalization of the statistical report. The primary endpoint will be analyzed using a log-logistic regression model with the treatment group as covariate. Results from the model will be presented as the difference in median time in hospital LOS, with 95% confidence intervals. Deaths will be imputed with worst outcome. Secondary dichotomous endpoints will be analyzed using logistic regression with the treatment group as covariate. Results from the model will be presented as odds ratios with 95% confidence intervals. Missing data will be imputed with worst outcome. Secondary continuous endpoints will be analyzed using linear regression for non-repeated measures or mixed effects linear regression for repeated measures. Generalized linear model versions or quantile regression models will be considered if the assumptions underlying the normal linear model are assumed to be violated based on blinded analyses. Treatment differences will be presented with 95% confidence intervals. Time to event endpoints will be analyzed using competing risk methodology, with the event and death as competing risks. Results from the model will be presented as hazard ratios with 95% confidence intervals.

### Data management

All data will be handled confidentially. Access to patient records will be limited to the study group. Patients will be pseudonymized by study identification numbers, and all data will be handled without using names or personal social security numbers. Some of the source data will be stored in a dedicated and secured area at Oslo University Hospital. Data will be stored in a de-identified manner, where each study participant is recognizable by his/her unique trial patient number. A username and password-protected web-based eCRF is generated for each participant (Viedoc™, PCG Solutions, S:t Persgatan 6, Uppsala, Sweden). The data will be stored for 5 years after the final report on the research project has been sent to the ethical committee. Permission for the database has been submitted to Oslo University Hospital, and a description of the database has been provided.

### Interim analyses

The MINIMUM study is not blinded. Consequently, the researches will follow each patient carefully regarding complications. Therefore, the interim analysis will analyze only safety data. An interim analysis will be performed after 50% of the patients have ended the 30-days follow-up visit. Data to be included within the interim analyses are:

- Hemorrhage with hemodynamic affection (development of circulatory shock or need of transfusion)
- Infections
- Deaths; all-cause mortality

Interim analysis is performed by the statistician allocated to this study accompanied by Project Manager/Coordinating Investigator and Academic responsible and supervisor in the MINIMUM study.

### Criteria for premature termination of a patient’s participation in the trial

Consent withdrawal or in the case of the surgery ending without a pancreatoduodenectomy being performed will be considered a termination of the patient’s participation in MINIMUM. A malfunction of the microdialysis catheter or analysis equipment will make it impossible to achieve further adequate microdialysis data. The patients will be followed until discharge and follow-up appointments at 30th and 90th POD, irrespective of a malfunctioning catheter or not.

### Criteria for termination of the clinical investigation

The study may be terminated by the Research responsible representative or the Project Manager/Coordinating Investigator at any time. However, scheduled follow-up, as described in Assessment Schedule, should be continued for all patients who were treated prior to termination of the study. The clinical investigation may be discontinued at the discretion of the Project Manager/Coordinating Investigator, the supervisor, or the Research responsible representative in the event of any of the following:

- Occurrence of adverse events unknown to date in respect of their nature, severity, and duration
- Medical or ethical reasons affecting the continued performance of the trial
- By the interim analysis group assessment
- Difficulties in the recruitment of patients which makes it impossible to include enough patients in an adequate time period.
- Cancelation/cessation of microdialysis catheter production which makes it impossible to acquire catheters.

The Research responsible representative and Project Manager/Coordinating Investigator will inform all investigators, local hospital authority, and the ethics committee of the termination of the trial along with the reasons for such action. If the study is terminated early on grounds of safety, the ethics committee will be informed within 15 days.

## Reporting adverse events

Adverse events will be reported in the local “Adverse Event Reporting system” at the including center and will be recorded in the eCRF. The adverse event report will follow standard assessment and management given at Oslo university hospital, i.e. an adverse event note will be posted by the investigator in the hospitals’ own quality system software (Achilles, CGI Norge AS, Oslo, Norway). Thereafter, experts will assess the incident, obtain additional expertise if necessary, and then perform changes (if applicable) in the hospital’s system for the purpose to avoid any later similar incidents. Additionally, any adverse event will be documented in the patient’s medical record and reported to the Project Manager/Coordinating Investigator by the principal investigator. The Project Manager/Coordinating Investigator and the principal investigator will then discuss whether the adverse event could have led to a serious adverse event. In case of disagreement between Project Manager/Coordinating Investigator and the principal investigator, the Project Manager / Coordinating Investigator shall communicate both opinions to the Research responsible The protocol may require representative, catheter manufacturer, and the ethics committee.

## Protocol amendments

The protocol may require amendments during the conduct of a clinical investigation. Any amendment to the protocol will be agreed upon between the Project Manager / Coordinating Investigator and the principal investigator. The amendments will be notified (in the case of non-substantial amendments) to, or approved by (in the case of substantial amendments) the ethics committee and local authority.

## Deviations from protocol

By signing the protocol, the investigator confirms that the clinical investigation will be performed in accordance with the protocol. A disqualification of an investigator or principal investigator from the clinical investigation may be applicable if mismanagement and neglect of following this protocol persist despite several feedbacks given by the Project Manager/Coordinating Investigator/Research responsible representative.

## Ethical considerations

All patients will follow standard surgical procedure at the site. The only addition is placing a microdialysis catheter near the anastomosis during the end of surgery in half of the patients. Sampling dialysate from the catheter is not associated with any discomfort. Blood samples will not be timewise in excess of routine blood testing. Project Manager / Coordinating Investigator and Research responsible representative will monitor/follow drop-outs, protocol deviations and perform a safety interim-analysis after 50% of patients have finished the study period. This means that if microdialysis method used in clinical assessments are particularly beneficial or harmful compared to the control group while the study is on-going, the Project Manager/Coordinating Investigator and Research responsible representative may consider termination of the study earlier than planned.

All patients with a microdialysis catheter and microdialysate value of glycerol ≥ 400 μmol/L in three consecutive samples will have an abdominal CT scan on POD 2. The radiation dose associated with this examination is considered negligible.

## Discussion

Pancreatic surgery is the only curative modality for pancreatic neoplasm. Despite better surgical techniques and improvement in postoperative care, the morbidity and mortality are still high. For the most part the morbidity is due to postoperative pancreatic fistula, which is regarded as the most threatening complication after pancreatic resection. This risk is also present for other diseases where a pancreatoduodenectomy is performed. Developing a POPF in a patient may have a great influence for the patient considering increased morbidity and mortality, but also may have a big impact for the hospital and the financial burden to the society. Diagnosing a POPF is traditionally based on clinical assessment together with blood samples and CT scans. These diagnostic tools reveal a POPF relatively late in the course of the natural development of this complication which may account for the high observed morbidity and mortality. Also, in a pancreatoduodenectomy an abdominal drain is inserted at the end of surgery and is commonly removed on POD 3. This can be a disadvantage in terms of fast mobilization after surgery. Using the microdialysis method the investigators are hoping to reveal a POPF at an earlier stage thereby initiate earlier interventions. Also, if microdialysis at POD 1 reveals no suspicion of a POPF, the abdominal drain is removed the POD 1, allowing for early mobilization. This in turn may reduce LOS at the hospital, morbidity, and mortality.

Therefore, the current trial will focus on generating information of how microdialysis results may impact the postoperative course after pancreatoduodenectomy. Both morbidity, LOS, costs, and any adverse effects will be assessed. Blinding of the study is not possible. The group affiliation is obvious for the different caregivers and the patients. Another limitation is that this study is a single-center study, potentially reducing the generalizability. As far as we know, this is the first randomized, controlled study to compare microdialysis versus standard of care in patients undergoing pancreatoduodenectomy. The study findings could improve the future treatment of patients undergoing pancreatoduodenectomy. Intraabdominal microdialysis could potentially improve the postoperative course reducing morbidity and mortality. Furthermore, there is a great potential for shortening ICU and hospital length of stay, and considerably reducing the cost for this group of patients. This study may open the possibility for using microdialysis as standard monitoring in patients undergoing pancreatoduodenectomy. Also, information about the patient course until 90 days postoperatively may reveal both short- and long-term follow-up knowledge. We are planning to publish our results in international peer-review journals and authorship will be awarded according to Vancouver convention.

## Trial status

Approval by the Ethics Committee at Oslo University Hospital was received 7th of September 2018 (reference 2018/1334/REK nord), and patient recruitment started in April 2019. By end of March 2020 116 patients are enrolled. Current protocol version is 19-1, dated 15th January 2019. Patient recruitment will be completed before mid-2021.

## Assessment Schedule

This article has been written according to the guidelines for content of clinical trial protocols (SPIRIT) with a checklist (Additional file 3) and an Assessment Schedule as shown in Table 2.

## Supplementary Information

**Additional file 1.**

**Additional file 2.**

**Additional file 3.**

**Additional file 4.**

**Additional file 5.**

**Additional file 6.**

## Data Availability

The data sets generated and/or analyzed during the current study are not publicly available due to Norwegian laws on privacy protection but are available from the corresponding author on reasonable request.

## Abbreviations

ALAT: Alanine transaminase
ALP: Alkaline phosphatase
ASA: American Society of Anesthesiologists
ASAT: Aspartate transaminase
BMI: Body mass index
BPmap: Mean arterial blood pressure
C: Celcius
CRP: C-reactive protein
CT: Computer tomography
eCRF: Electronic case report form
GGT: Gamma-glutamyltransferase
GFR: Glomerular filtration rate
Hb: Hemoglobin
ICU: Intensive care unit
INR: International normalized ratio
iv: Intravenous
LOS: Length of stay
LD: Lactate dehydrogenase
L:P: lactate:pyruvate
MRI: Magnetic resonance imaging
POPF: Postoperative pancreatic fistula
PWB: Predicted body weight
PD: Pancreatic duct
POD: Postoperative day
QoL: Quality of life
SF-MPQ: Short-form McGill Pain Questionnaire
RPM: Rounds per minute
SpO2: Peripheral oxygen saturation
SOFA: Sequential Organ Failure Assessment
TNM: Tumor Node Metastasis
Trc: Thrombocytes
WBC: White blood cells
