# Supplementary material for: Monitoring of patients with microdialysis following pancreaticoduodenectomy—the MINIMUM study: study protocol for a randomized controlled trial
Source: Trials. 2021 May 7;22:329. doi: 10.1186/s13063-021-05221-9 (PMC8105916; doi:10.1186/s13063-021-05221-9)
Supplement: Supplementary file 1 — Additional file 1. [file 13063_2021_5221_MOESM1_ESM.docx]

# Appendix 1 - Funding documentation MINIMUM Trial

## E-mail from South-Eastern Norway Regional Health Authority to chief of research group, professor Tor Inge Tønnessen. Total amount is 9 mill Norwegian Kroner.

**Fra:** Berit Merete Øien <[Berit.Merete.Oien@helse-sorost.no](mailto:Berit.Merete.Oien@helse-sorost.no)>
**Dato:** torsdag 17. desember 2015 15:23
**Til:** Tor Inge Tønnessen <[t.i.tonnessen@medisin.uio.no](mailto:t.i.tonnessen@medisin.uio.no)>
**Emne:** Tildeling av forskningsmidler for 2016 fra Helse Sør-Øst

**Kjære Tor Inge Tønnessen**

Prosessen med tildeling av forskningsmidler for 2016 er nå avsluttet. Det ble behandlet i alt 536 søknader.

Vi gratulerer deg med tildeling av forskningsmidler til prosjektet/forskningstiltaket:

Søknad:                                               *Implantable sensors for real-time detection of organ pathology; Multicenter studies*

Prosjektleder (søker):                  Tor Inge Tønnessen

Søknadstype:                                   Åpen prosjektstøtte

                Tildelt:  **Åpen prosjektstøtte**

Søknadskategori:                            Klinisk somatisk forskning, inkl. translasjonsforskning

Helseforetak/sykehus/

Institusjon                                         **Oslo universitetssykehus HF**

Prosjektet har fått tildelt prosjekt nr.: **2016115**

**Prosjektnummeret skal brukes ved all kommunikasjon med Helse Sør-Øst RHF.**

Prosjektvarighet:            3 år/100 %

Oppstartsdato:                1/1/2016

Dersom prosjektets oppstartsdato endres må vi ha melding om dette. Send e-post til [forskningsmidler@helse-sorost.no](mailto:forskningsmidler@helse-sorost.no).

**Siste frist for oppstart av prosjektet er 1. desember 2016.**

Se våre retningslinjer vedrørende endringer i tildelte prosjekter på våre nettsider: <http://www.helse-sorost.no/fagfolk_/forskning_/forskningsmidler_/Sider/endringer-i-pagaende-prosjekter.aspx>

Dersom tildelingen gjelder vitenskapelige stillinger uten navngitt kandidat, skal navn meldes inn til Helse Sør-Øst straks dette er klart. Send e-post til [forskningsmidler@helse-sorost.no](mailto:forskningsmidler@helse-sorost.no)

Midlene utbetales til søkerinstitusjon (Oslo universitetssykehus HF) og er knyttet til  (”øremerket”) prosjektet.

Tildelt for 2016:                                3000000

Tilsagn for øvrige år:

Tilsagn for 2017:                              3000000

Tilsagn for 2018:                              3000000

Tilsagn for 2019:

Tilsagn for 2020:

Tilsagn for 2021:

Tilsagn for 2022:

Tilsagn for 2023:

Før overføring av midler kan skje må du bekrefte om du ønsker å motta forskningsmidlene.  Vi ber derfor om at du besvarer denne e-posten innen 7. januar 2016 til [berit.oien@helse-sorost.no](mailto:berit.oien@helse-sorost.no)

Det er tilstrekkelig at du besvarer følgende spørsmål:

1. ***JA, Jeg takker ja til forskningsmidlene til prosjektnr.:  2016115 og bekrefter samtidig at jeg ikke mottar eller kommer til å motta finansiering av samme tiltak fra annen finansieringskilde (”dobbeltfinansiering”).^[[1]](#footnote-1)^[1]***
2. ***NEI, Jeg frafaller midlene til prosjektnr.: 2016115***

¹ I de tilfellene hvor Helse Sør-Øst ikke dekker hele prosjektkostnaden kan man motta delfinansiering fra andre kilder innenfor budsjettet i opprinnelig søknad.

Ytterligere informasjon om tildeling av forskningsmidler for 2016 finner du på våre nettsider, [www.helse-sorost.no](http://www.helse-sorost.no)  samt i [styresak 085-2015](http://www.helse-sorost.no/aktuelt_/aktiviteter_/Sider/Styremøte-17.-desember-2015.aspx)

Vennlig hilsen

Avdeling for forskning og innovasjon

Helse Sør-Øst RHF

Øystein Krüger                                                                               Berit Merete Øien

forskningssjef                                                                                 rådgiver

1. [↑](#footnote-ref-1)
