# Supplementary material for: Monitoring of patients with microdialysis following pancreaticoduodenectomy—the MINIMUM study: study protocol for a randomized controlled trial
Source: Trials. 2021 May 7;22:329. doi: 10.1186/s13063-021-05221-9 (PMC8105916; doi:10.1186/s13063-021-05221-9)
Supplement: Supplementary file 2 — Additional file 2. [file 13063_2021_5221_MOESM2_ESM.docx]

# Appendix 2 - Funding documentation MINIMUM Trial

## E-mail from South-Eastern Norway Regional Health Authority to chief of research group, professor Tor Inge Tønnessen. Total amount is 9 mill Norwegian Kroner.

**From:** Berit Merete Øien <[Berit.Merete.Oien@helse-sorost.no](mailto:Berit.Merete.Oien@helse-sorost.no)>
**Date:** Thursday 17. Desember 2015 15:23
**To:** Tor Inge Tønnessen <[t.i.tonnessen@medisin.uio.no](mailto:t.i.tonnessen@medisin.uio.no)>
**Subject:** Confirmation of research funding, 2016 from South-Eastern Norway Regional Health Authority

**Dear Tor Inge Tønnessen**

We congratulate you on the allocation of research funding to the project / research initiative:

Application:                                     *Implantable sensors for real-time detection of organ pathology; Multicenter studies*

Project Manager (applicant):      Tor Inge Tønnessen

Application group: Open project support

Allocation Open project support

Allocation category: Clinical somatic research, including translational research

Trust/ Hospital /Institution: Oslo University Hospital HF

The project has been awarded project no.: **2016115**

**The project number must be used for all communication with South-Eastern Norway Regional Health Authority**

Project duration: 3 years/100 %

Starting date: Jan. 1^st^ 2016

If the project start date changes, we must notify. Send email to [forskningsmidler@helse-sorost.no](mailto:forskningsmidler@helse-sorost.no).

**The deadline for starting the project is 1^st^ December 2016.**

See our policy on changes to assigned projects on our website: <http://www.helse-sorost.no/fagfolk_/forskning_/forskningsmidler_/Sider/endringer-i-pagaende-prosjekter.aspx>

If the award applies to scientific positions without an unnamed candidate, names must be reported to South-Eastern Norway Regional Health Authority as soon as this is clear. Send email to [forskningsmidler@helse-sorost.no](mailto:forskningsmidler@helse-sorost.no)

The funds are paid to the applicant institution (Oslo University Hospital HF) and are linked to the project.

Awarded for 2016:                                3000000

Awarded for former years:

Awarded for 2017:                              3000000

Awarded for 2018:                              3000000

Awarded for 2019:

Awarded for 2020:

Awarded for 2021:

Awarded for 2022:

Awarded for 2023:

Before funds can be transferred, you must confirm whether you want to receive the research funding. We therefore request that you reply to this email by January 7^th^, 2016 [berit.oien@helse-sorost.no](mailto:berit.oien@helse-sorost.no)

It is sufficient that you answer the following questions:

1. ***• YES, I accept the research funding for project no .: 2016115 and at the same time confirm that I do not receive or will receive funding for the same initiative from another source of funding ("double funding"). ^[^1]***
2. ***NO, I refuse the funds for project no .: 2016115***

¹ In cases where South-Eastern Norway Regional Health Authority does not cover the entire project cost, you can receive partial funding from other sources within the budget in the original application.

Further information on the allocation of research funding for 2016 can be found on our website, [www.helse-sorost.no](http://www.helse-sorost.no)  and [styresak 085-2015](http://www.helse-sorost.no/aktuelt_/aktiviteter_/Sider/Styremøte-17.-desember-2015.aspx)

Best regards

 Departmenet for research and innovation

South-Eastern Norway Regional Health Authority

Øystein Krüger                                                                               Berit Merete Øien

Chief of research                                                                               Counsellor
