# Supplementary material for: Monitoring of patients with microdialysis following pancreaticoduodenectomy—the MINIMUM study: study protocol for a randomized controlled trial
Source: Trials. 2021 May 7;22:329. doi: 10.1186/s13063-021-05221-9 (PMC8105916; doi:10.1186/s13063-021-05221-9)
Supplement: Supplementary file 3 — Additional file 3. [file 13063_2021_5221_MOESM3_ESM.pdf]

**Region:**	**Saksbehandler:**	**Telefon:**	**Vår dato:**	**Vår referanse:**
REK nord			07.09.2018	2018/1334/REK nord
			**Deres dato:**	**Deres referanse:**
			12.06.2018	

Vår referanse må oppgis ved alle henvendelser

Espen Lindholm
Postboks 4950 Nydalen

2018/1334 Monitorering med mikrodialyse etter Whipple operasjon - en randomisert studie

Forskningsansvarlig institusjon: Oslo universitetssykehus HF, St. Olavs Hospital HF
Prosjektleder: Espen Lindholm

Vi viser til søknad om forhåndsgodkjenning av ovennevnte forskningsprosjekt. Søknaden ble behandlet av Regional komité for medisinsk og helsefaglig forskningsetikk (REK nord) i møtet 23.08.2018. Vurderingen er gjort med hjemmel i helseforskningsloven (hfl.) § 10.

Prosjektleders prosjektomtale
Ved kreft i bukspyttkjertelen gjøres en Whipple operasjon der deler/hele bukspyttkjertelen fjernes. Den vanligste og alvorligste komplikasjonen er lekkasje mellom tarm og bukspyttkjertel-rest som forekommer i 20-30% av operasjonene. Resultatet er ofte lange sykehus- og intensivopphold, reoperasjoner og død (3-5%). Å oppdage en lekkasje tidlig i forløpet før pasienten blir alvorlig syk for å igangsette behandling er derfor meget viktig. Vi ønsker å legge inn et tynt plastkateter (mikrodialysekateter) nær operasjonsområdet i buken. I dette kateteret gjør vi mikrodialyse slik at stoffer som melkesyre, pyruvat, glyserol m.fl. fra skjøteområdet kan analyseres. Hvis en pasient har lekkasje, vil glyserol stige kraftig og melkesyre- og pyruvatverdiene være annerledes enn hos pasienter som ikke har lekkasje. Vi tror at å analysere slik, kan man oppdage lekkasje på et tidlig tidspunkt før situasjonen blir alvorlig og dermed unngå høy sykelighet og død. Vi ønsker å gjøre en randomisert studie.

Om prosjektet
Dette er en 2-armet, multisenter, randomisert, parallellgruppe kontrollert studie (RCT) som gjennomføres ved OUH og St. Olav Hospital hos pasienter som gjennomgår en Whipple operasjon der deler/hele bukspyttkjertelen skal fjernes. Man tar sikte på å inkludere 200 pasienter, hvorav halvparten av de inkluderte pasientene vil motta et intraperitonealt mikrodialysekateter implantert i form av et tynt plastkateter (mikrodialysekateter) nær operasjonsområdet i buken. I dette kateteret vil man gjøre mikrodialyse, slik at stoffer som melkesyre, pyruvat, glyserol m.fl. fra skjøteområdet kan analyseres. Hvis en pasient har lekkasje, vil glyserol stige kraftig og melkesyre- og pyruvatverdiene være annerledes enn hos pasienter som ikke har lekkasje.

Det tas blodprøver og det er lagt opp til kontaktpunkter underveis for å fange opp signaler på lekkasje.

Formålet er å finne en metode for å oppdage en lekkasje tidlig, slik at man unngår de vanligste og alvorligste komplikasjonen som lekkasje mellom tarm og bukspyttkjertel-rest medfører.

Forskningsbiobank
Det søkes om å opprette en spesifikk forskningsbiobank med navn MINIMUM og vil være lokalisert ved OUS - Rikshospitalet.

Ansvarlig for biobanken vil være Espen Lindholm.

Forskningsbiobanken vil bestå av serum, plasma, kroppsvæsker, annet materiale som mikrodialyse og drensvæske fra buk

Forespørsel/informasjonsskriv/samtykkeerklæring
Det er ikke noe prinsipielt i veien for å ha med et bilde i informasjonsskrivet, men slik informasjonsskrivet ser ut nå, tar bildet opp en del av plassen som burde vært forbeholdt annen essensiell informasjon. For eksempel så kommer det først med på side 2 informasjon om randomisering. «*Det er viktig å poengtere at pasientene deles i 2 like store grupper. Det er loddtrekning og helt tilfeldig om du havner i det ene eller andre gruppen: 1) En gruppe som får mikrodialysekateter og man bruker mikrodialyse-analysene som tilleggsopplysninger i forløpet etter operasjon som et ekstra hjelpemiddel for å behandle en ev. lekkasje mellom bukspyttkjertel og tarm. 2) En gruppe som ikke får mikrodialysekateter og som overvåkes og behandles etter de nåværende gjeldende retningslinjer i forløpet av en slik type operasjon*.» Denne informasjonen bør komme tidlig i informasjonsskrivet.

Informasjon om hvem som en ansvarlig og hvilket foretak som står for studien er viktig informasjon, men ikke essensiell og bør derfor flytes lengre bak i skrivet.

Det fremgår av søknaden at både biologisk materiale og helseopplysninger skal tas ut av Norge. Dette må også fremkomme av informasjonsskrivet.

Det står også at «*Prøvene vil også kunne lagres i biobanken etter studiens slutt til bruk i senere studier. Du gir herved anledning for forskerne å gjøre dette. Blodprøvene vil ikke overlates til andre forskere uten nytt samtykke fra deg.*»

Komiteen vil gjøre prosjektleder oppmerksom på at dersom det biologiske materialet skal overføres til generell biobank etter prosjektsslutt, må det lages et avkrysningsfelt for dette og det må gis tilstrekkelig informasjon om dette for at samtykket skal anses dekkende, eventuelt kan man legge ved skrivet for den generell biobanken, slik at deltagerne kan samtykke til begge deler samtidig.

Komiteen forutsetter at informasjonsskriv knyttet til studien revideres i tråd med ny mal på REKs nettsider, slik at informasjonen som gis til deltakerne er forenlig med ny personopplysningslov.

Vedtak
REK har gjort en helhetlig forskningsetisk vurdering a alle prosjektets sider og godkjenner det med hjemmel i helseforskningsloven § 10. Vi gjør samtidig oppmerksom på at etter ny personopplysningslov må det også foreligge et behandlingsgrunnlag etter personvernforordningen. Det må forankres i egen institusjon.

Før prosjektet kan igangsettes må det sendes inn revidert informasjonsskriv. Skrivet sendes som vedlegg i e-post til post@helseforskning.etikkom.no

Sluttmelding og søknad om prosjektendring
Prosjektleder skal sende sluttmelding til REK nord på eget skjema senest 01.05.2023, jf. hfl. § 12. Prosjektleder skal sende søknad om prosjektendring til REK nord dersom det skal gjøres vesentlige endringer i forhold til de opplysninger som er gitt i søknaden, jf. hfl. § 11.

Klageadgang
Du kan klage på komiteens vedtak, jf. forvaltningsloven § 28 flg. Klagen sendes til REK nord. Klagefristen

er tre uker fra du mottar dette brevet. Dersom vedtaket opprettholdes av REK nord, sendes klagen videre til Den nasjonale forskningsetiske komité for medisin og helsefag for endelig vurdering.

Med vennlig hilsen

May Britt Rossvoll
sekretariatsleder

Kopi til:line@ous-hf.no; per.einar.uggen@stolav.no
