# Supplementary material for: Monitoring of patients with microdialysis following pancreaticoduodenectomy—the MINIMUM study: study protocol for a randomized controlled trial
Source: Trials. 2021 May 7;22:329. doi: 10.1186/s13063-021-05221-9 (PMC8105916; doi:10.1186/s13063-021-05221-9)
Supplement: Supplementary file 4 — Additional file 4. [file 13063_2021_5221_MOESM4_ESM.docx]

**2018/1334 Microdialysis monitoring after Whipple surgery - a randomized study**

**Responsible research institution: Oslo University Hospital HF, St. Olavs Hospital HF**

**Project Manager: Espen Lindholm**

We refer to the application for approval of the above research project. The application was processed by the Regional Committee for Medical and Health Research Ethics (REK nord) at a meeting 23.08.2018. The assessment is made on the basis of § 10 of the Health Research Act.

**From the Project manager review**

In pancreatic cancer, a Whipple operation is performed where parts / entire pancreas is removed. The most common and most serious complication is a leakage between the intestine and the residual pancreas which occurs in the 20-30% of the operations. The result is often long hospital and ICU length of stay, reoperations, and deaths (3-5%). Detecting early a leak before the patient becomes seriously ill and to initiate treatment is therefore particularly important. We want to insert a thin plastic catheter (microdialysis catheter) near the operating area in the abdomen. In this catheter we perform microdialysis so that substances such as lactic acid, pyruvate, glycerol, and other metabolites from the anastomosis can be analyzed. If a patient has a leak, glycerol will rise sharply, and lactic acid and the pyruvate values ​​may be different than in patients who do not have a leak. We believe that analyzing this way will detect leakage at an early stage before the situation becomes severe and thus avoid high morbidity and death. We want to perform a randomized study.

**About the project**

This is a 2-arm, multicenter, randomized, parallel group-controlled trial (RCT) conducted at OUH and St. Olav Hospital in patients undergoing a Whipple surgery where parts / whole of the pancreas is removed. The aim is to include 200 patients, half of the included patients will receive an intraperitoneal microdialysis catheter implanted in the form of a thin plastic catheter (microdialysis catheter) near the abdominal operating area. In this catheter, microdialysis will be performed so that substances such as lactic acid, pyruvate, glycerol, and other metabolites from the anastomosis can be analyzed. If a patient has a leakage, glycerol will rise sharply, and lactic acid and pyruvate values will be different than in patients who do not have leakage.

Blood samples are taken and visiting points are set up along the way to detect signals of leakage.

The aim is to find a method for detecting a leakage early, to avoid the most common and severe complication that is leakage in the anastomosis between intestine and the pancreas remains.

**Research Biobank**

An application is submitted to create a specific research biobank named MINIMUM and will be located at OUH - Rikshospitalet.

Responsible for the biobank will be Espen Lindholm.

The research biobank will consist of serum, plasma, body fluids, other materials such as microdialysis and drainage fluid from the abdomen.

**Inquiry / information sheet / consent form**

There is nothing wrong by including a picture in the information letter, but as it appears now, the picture takes up part of the space that should have been reserved for other essential information. For example, first at page 2 the information about randomization occur. *"It's important to point that out that the patients are divided into 2 equal groups. It is a kind of lottery and totally random whether you end up in one or second group: 1) A group that receives a microdialysis catheter and one uses the microdialysis assays as additional information in the process after surgery as an additional aid for treating an leakage between the pancreas and intestine. 2) A group that does not receive microdialysis catheters and is monitored and are treated according to the current local guidelines at the hospital for such a type of operation. "* This kind of information should come early in the information letter.

Information about who is responsible and which company is responsible for the study is important information, however not essential and should therefore be placed further behind in this information sheet.

The application states that both biological material and health information can be brought out of Norway. This must also be stated in the information letter.

It also states that “*The samples may also be stored in the biobank after the end of the study for use in later studies. You give researchers the opportunity to do this. The blood tests will not be given to other researchers without new consent from you.*”

The committee will make the project manager aware of that if the biological material is to be transferred to a general biobank after the project end, a check box must be created for this and sufficient information must be provided for the consent to be considered comprehensive, otherwise, you can enclose another written consent for a general biobank, so that participants can consent to both at the same time.

The committee assumes that the information sheet used for the study is in line with a new template on EC's website, so that the information given to participants is compatible with new General Data Protection Regulation.

**Decision**

The EC has made a comprehensive research ethics assessment on all sides of the project and approved it based on the §10 of the Health Research Act. We also note that the research must be based on a need for treatment according to the new General Data Protection Regulation. This must be embedded in your own institution.

Before the project can be started, a revised information letter must be submitted. The letter is sent as an attachment in an e-mail to [post@helseforskning.etikkom.no](mailto:post@helseforskning.etikkom.no)

**Final notification and application for project amendments**

The project manager shall send a final report to REK nord on their own form no later than 01.05.2023, in line with the Health Research Act. §12. The project manager shall send an application for significant changes in the project compared to the provided information in the application, in line with Health Research Act § 11.

**Right to appeal**

You can appeal the committee's decision, in line with the Public Administration Act § 28. The complaint is sent to REK nord. The deadline for appeal is three weeks from you receive this letter. If the decision is sustained by REK nord, the complaint is forwarded to The National Research Ethics Committee for Medicine and Health Sciences for final assessment.

With best regards

May Britt Rossvoll

Head of secretariat

**Copy to**: line@ous-hf.no; per.einar.uggen@stolav.no
