# Supplementary material for: Monitoring of patients with microdialysis following pancreaticoduodenectomy—the MINIMUM study: study protocol for a randomized controlled trial
Source: Trials. 2021 May 7;22:329. doi: 10.1186/s13063-021-05221-9 (PMC8105916; doi:10.1186/s13063-021-05221-9)
Supplement: Supplementary file 6 — Additional file 6. [file 13063_2021_5221_MOESM6_ESM.docx]

# Forespørsel om deltakelse i forskningsprosjektet

**Tidlig oppdagelse av bukspyttlekkasje ved bruk av Mikrodialyse etter bukspyttkjerteloperasjon**

**THE MIMINUM STUDY**

Du inviteres med dette til deltakelse i et forskningsprosjekt fordi du skal gjennomgå en bukspyttkjerteloperasjon. Forskningsprosjektet er igangsatt av Akuttklinikken, Anestesiologisk avdeling ved Oslo Universitetssykehus (OUS). Vi ønsker å undersøke om metoden mikrodialyse kan bidra til tidligere oppdagelse av lekkasje i bukspyttkjertelen etter gjennomgått bukspyttkjerteloperasjon (såkalt Whippel-operasjon). Risiko for en slik bukspyttkjertellekkasje ligger statistisk på ca. 10-20%. Før du bestemmer deg om du vil delta, les dette informasjonsskrivet nøye. Be også om å få besvart alle spørsmål du har om deltagelse i denne studien.

## Hva innebærer PROSJEKTET?

Pasientene deles i 2 like store grupper. Det er loddtrekning og helt tilfeldig om du havner i det ene eller andre gruppen:

1. En gruppe som får mikrodialysekateter og man bruker mikrodialyse-analysene som tilleggsopplysninger i forløpet etter operasjon som et ekstra hjelpemiddel for å behandle en ev. lekkasje mellom bukspyttkjertel og tarm.
2. En gruppe som ikke får mikrodialysekateter og som overvåkes og behandles etter de nåværende gjeldende retningslinjer i forløpet av en slik type operasjon.

Denne metodikken er en god metode for å finne ut om resultatene man får ved mikrodialyseteknikk er bedre enn dagens standard for behandling. I alt planlegges det å inkludere 200 pasienter.

På slutten av operasjonen vil halvparten av forsøkspersonene få plassert et tynt plastrør (mikrodialysekateter) festet i nærhet til der restene av bukspyttkjertelen blir operert inn på tarmen. Kateteret blir liggende ut i huden hvor det kobles til en pumpe som pumper en væske (mikrodialysatet) gjennom kateter. Det foregår så en såkalt mikrodialyse der avfallsstoffer fra operasjonsområdet siver inn i denne væsken som så pumpes tilbake i en beholder. Væsken i denne beholderen kan så analyseres for stoffer som kan gi mistanke om det foreligger betennelse eller lekkasje i tarmen etter gjennomført operasjon. Formålet med studien er å finne ut om denne metodikken kan gjøre at kirurgen kan oppdage en lekkasje på et tidligere tidspunkt enn hvis kirurgen ikke visste om disse analysene. Dermed kan man også behandle lekkasjen på et tidligere tidspunkt og sannsynligvis redusere komplikasjoner etter operasjonen i tillegg til at rehabiliteringen går rasker.

Før operasjon vil du få en samtale med utprøver der man vil notere ned tidligere sykehistorie, medikamentbruk, gjøre en fysisk undersøkelse, registrere resultat av prøver og undersøkelser gjort før operasjonen om aktuell sykdom samt be deg fylle ut to spørreskjemaer om livskvalitet og smerte. All informasjon hentes fra undersøkelser, intervju med deg samt pasientjournal. Det vil bli tatt blodprøver før operasjon og disse vil sammenliknes med blodprøver tatt etter operasjon. Selve operasjonen er uendret i forhold til vanlig rutine på sykehuset med unntak av dette mikrodialysekateteret. Kateteret vil oppleves som lite plagsomt. Du vil også kunne ha andre plastslanger ut i fra huden på magen (som er rutine ved slike operasjoner), slik at dette er ikke spesielt annerledes enn ordinær praksis. Første døgnet etter operasjon vil du bli liggende på postoperativ seksjon for observasjon. Blod- og mikrodialysatprøver vil bli tatt daglig også seinere på sykehusoppholdet. Det vil bli registrert daglig væskeinntak, organfunksjon og ev. komplikasjoner så lenge du er på sykehuset. Spørreskjemaene tilsvarende før operasjon vil bli gjentatt dag 3 etter operasjon og ved avreise fra sykehuset. Før avreise fra sykehuset vil mikrodialysekateteret fjernes ved at man trekker det ut fra huden. Denne prosedyren vil ikke medføre smerter eller ubehag.

Hos de som har fått mikrodialysekateter og som samtidig ut i fra mikrodialyse-analysene tyder på en mulig lekkasje fra bukspyttkjertel, vil du dag 2 etter operasjonen få gjennomført en røntgenundersøkelse av magen din, en såkalt CT der man vil se om bildene kan avsløre en ev. lekkasje. Hvis det foreligger en lekkasje, vil kirurgen igangsette forskjellige behandlingsmuligheter på et raskere tidspunkt enn det er mulig uten mikrodialysekateter. For øvrig vil man følge vanlig rutine ved sykehuset ved disse operasjonene. Du vil også bli ringt til 30 og 90 dager etter operasjonen for å spørre hvordan det går og om du har vært innlagt på sykehus siden siste utskrivelse. Her vil man også be om at det fylles ut de samme to spørreskjemaer.

Hovedansvarlig for studien er Espen Lindholm, lege og forsker ved Akuttklinikken, Avd. for anestesiologi, OUS. Studien vil foregå på flere steder:

Oslo Universitetssykehus – Rikshospitalet

St. Olav Hospital HF

Ansvarlig ved ditt sykehus vil være: …….., Overlege Anestesiologisk avdeling, ……...

## Mulige fordeler og ulemper

Du vil i denne studien følge gjeldende klinisk praksis med hensyn til kirurgisk inngrep. Mikrodialysekateteret som du vil ha mens du er inneliggende vil erfaringsvis ikke gi deg noen spesielle plager. På den 2. postoperative dag utføres en CT undersøkelse (røntgen) hos de pasienter som har mikrodialysekateter og samtidig mikrodialyse-analyser som kan tyde på en lekkasje. Med moderne CT maskiner er det i dag en veldig lav stråledose, og stråledosen kan sammenlignes med stråling vi måler med konvensjonelle røntgenundersøkelser. Det er mulig å få en allergisk reaksjon på kontrastmiddel gitt under CT-undersøkelsen. Dette er en ekstremt sjelden komplikasjon og vil bli håndtert i henhold til sykehusets rutiner. Pasienter som ikke ønsker å være deltaker i studien vil få ordinær behandling som man rutinemessig gir på sykehuset ved denne tilstanden.

I dag vet vi ikke om analyseresultater fra mikrodialyse kan være en hjelp for kirurgen eller ikke. Studien vil kunne gi svar på om det kan være en fordel å vite resultatene fra mikrodialysen slik at kirurgen eventuelt kan gjøre endringer i behandlingsplanen som kan gagne pasienten.

Uventede risikoer kan også forekomme. Det er derfor viktig at du forteller studiepersonalet om alt ubehag du opplever. Dersom det i løpet av studien skulle fremkomme ny informasjon eller endringer til prosjektet som kan påvirke ditt valg om å være med, vil du bli informert.

## Alternativ behandling

Hvis du ikke ønsker å være med i studien vil du få standard behandling som man vanligvis utfører ved denne type tilstand som du lider av. Du vil ikke få operert inn et mikrodialysekateter.

## Frivillig deltakelse og mulighet for å trekke sitt samtykke / tidlig avslutning

Det er frivillig å delta i prosjektet. Hvis du ønsker å delta, undertegner du samtykkeerklæringen på siste side. Du kan når som helst og uten å oppgi noen grunn trekke ditt samtykke. Dette vil ikke få konsekvenser for din videre behandling. Hvis du trekker deg fra prosjektet, kan du kreve å få slettet innsamlede prøver og opplysninger, med mindre opplysningene allerede er inngått i analyser eller brukt i vitenskapelige publikasjoner. Hvis du ønsker å trekke deg fra studien etter at du har startet din deltagelse, eller du har spørsmål til prosjektet, kan du kontakte

……… (Hovedutprøver, – avd. for ……., sykehus), tlf: XXXXXXXX, e-post: XXXXXX@xx.no

Legen din eller forskningsansvarlig kan avslutte din deltagelse i studien før den er ferdig hvis den ikke lenger anses som forsvarlig.

## Hva skjer med informasjonen om deg?

Din deltagelse i studien blir behandlet konfidensielt. Informasjonen som registreres om deg skal kun brukes slik som beskrevet i hensikten med studien. Du har rett til innsyn i hvilke opplysninger som er registrert om deg og rett til å få rettet eventuelle feil i de opplysningene som er registrert. Du har også rett til å få innsyn i sikkerhetstiltakene ved behandling av opplysningene.

Alle opplysningene vil bli behandlet uten navn og fødselsnummer eller andre direkte gjenkjennende opplysninger. En kode knytter deg til dine opplysninger gjennom en navneliste. Det er kun

OUS: Stipendiat Nil Ekiz, prosjektleder Espen Lindholm og faglig ansvarlig, Tor Inge Tønnessen (OUS) som har tilgang til denne listen i tillegg Per Einar Uggen ved St. Olavs hospital.

Prosjektleder har ansvar for den daglige driften av forskningsprosjektet og at opplysninger om deg blir behandlet på en sikker måte. Informasjon om deg vil bli anonymisert eller slettet 5 år etter prosjektslutt.

Representanter fra OUS og kontrollmyndigheter kan få utlevert studieopplysninger og gis innsyn i relevante deler av din journal. Formålet er å kontrollere at studieopplysningene stemmer overens med tilsvarende opplysninger i din journal. Alle som får innsyn i informasjon om deg har taushetsplikt.

Forskningsresultatene fra studien vil bli offentliggjort i vitenskapelige tidsskrift. Det vil ikke være mulig å identifisere deg i en slik offentliggjøring av resultater.

## Hva skjer med prøver som blir tatt av deg?

Prøvene som tas av deg skal oppbevares i en forskningsbiobank, kalt MINIMUM og vil være lokalisert ved OUS - Rikshospitalet. Ansvarlig for biobanken vil være Espen Lindholm. Blodprøvene som skal tas er

- Hemoglobin, blodplater, hvite blodlegemer
- Elektrolytter
- Nyrefunksjonsprøver
- Leverfunksjonsprøver
- Betennelsesmarkører som CRP og et panel av spesielle betennelsesstoffer
- Procalcitonin
- Lipase, amylase og bilirubin
- Blodgassanalyser

Forøvrig vil det også fryses ned mikrodialysevæske for analyse av betennelsesmarkører. Alle blodprøver for analyse av spesielle betennelsesstoffer skal fryses ned og sendes til OUS der de vil bli analysert av forskere ved OUS. Blodprøvene vil ikke overlates til andre forskere uten nytt samtykke fra deg. Biobanken opphører ved prosjektslutt.

## Forsikring

I Norge er det ingen spesiell forsikring tilknyttet studien, men alle pasienter er tilknyttet Norsk Pasientskade Erstatning (NPE) som ordinær praksis ved norske sykehus. Dersom en skade oppstår som følge av din deltagelse i prosjektet vil du etter egen vurdering fra NPE kunne få erstatning.

## ØKONOMI

Studien er finansiert gjennom forskningsmidler Helse Sør-Øst i Norge. Deltakerne vil ikke få noen økonomisk kompensasjon for deltakelse i studien.

## Utlevering av opplysninger til andre

Ved å delta i prosjektet, samtykker du også til at alle opplysninger som samles inn i forbindelse med studien kan utleveres til den aktuelle forskergruppen ved OUS-Rikshospitalet som er ansvarlig for studien.

## Godkjenning

Prosjektet er godkjent av Regional komite for medisinsk og helsefaglig forskningsetikk, saksnr. hos REK 2018/1334. Etter ny personopplysningslov har behandlingsansvarlig; OUS og St. Olavs Hospital og prosjektleder Espen Lindholm et selvstendig ansvar for å sikre at behandlingen av dine opplysninger har et lovlig grunnlag. Dette prosjektet har rettslig grunnlag i EUs personvernforordning artikkel 6a og 9a.

Du har rett til å klage på behandlingen av dine opplysninger til Datatilsynet.

## Kontaktinformasjon

Dersom du har spørsmål til prosjektet kan du ta kontakt med

OUS:

- Nil Ekiz (stipendiat og hovedutprøver), Tlf: 23 07 43 06 / 47 29 29 33, E-post: [nileki@ous-hf.no](mailto:nileki@ous-hf.no)
- Espen Lindholm (Prosjektleder), Tlf: 33 34 28 22 / 23 07 43 28 / 92 21 33 46, E-post: [line@ous-hf.no](mailto:line@ous-hf.no)
- Tor Inge Tønnessen (faglig ansvarlig), Tlf: 23 07 01 00 / 91 70 07 17, E-post: [ttonness@ous-hf.no](mailto:ttonness@ous-hf.no)

St. Olav Hospital:

- Per Einar Uggen (hovedutprøver), Tlf: 72 82 59 79/ 91 36 64 14, E-post: [per.einar.uggen@stolav.no](mailto:per.einar.uggen@stolav.no)

Du kan ta kontakt med institusjonens personvernombud dersom du har spørsmål om behandlingen av dine personopplysninger i prosjektet.

OUS: Personvernombudet OUS, Tlf: 02770 og e-postadresse: [personvern@ous-hf.no](mailto:personvern@ous-hf.no?body=Ikke%20skriv%20sensitive%20personopplysninger%20i%20e-post%20til%20sykehuset.)

St. Olav: Personvernombudet St. Olav Hospital, Tlf: 72 57 30 00, [post@stolav.no](mailto:post@stolav.no)

# Samtykke til deltakelse i PROSJEKTET

## Jeg er villig til å delta i prosjektet

| Sted og dato | Deltakers signatur |
| --- | --- |
|  |  |
|  | Deltakers navn med trykte bokstaver |

Jeg bekrefter å ha gitt informasjon om prosjektet

| Sted og dato | Signatur |
| --- | --- |
|  |  |
|  | Rolle i prosjektet |
